# Severe Malarial Thrombocytopenia: A Risk Factor for Mortality in Papua, Indonesia

**DOI:** 10.1093/infdis/jiu487

**Published:** 2014-08-28

**Authors:** Daniel A. Lampah, Tsin W. Yeo, Michael Malloy, Enny Kenangalem, Nicholas M. Douglas, Donny Ronaldo, Paulus Sugiarto, Julie A. Simpson, Jeanne Rini Poespoprodjo, Nicholas M. Anstey, Ric N. Price

**Affiliations:** 1 Timika Malaria Research Program, Papuan Health and Community Development Foundation; 2 Mimika District Health Authority; 3 Rumah Sakit Mitra Masyarakat, Timika, Papua; 4 Department of Pediatrics, Faculty of Medicine, Gadjah Mada University, Yogyakarta, Indonesia; 5 Global and Tropical Health Division, Menzies School of Health Research and Charles Darwin University; 6 Division of Medicine, Royal Darwin Hospital, Darwin; 7 Centre for Epidemiology and Biostatistics, Melbourne School of Population and Global Health, University of Melbourne; 8 Victorian Cytology Service, Carlton, Australia; 9 Lee Kong Chian School of Medicine, Nanyang Technological University; 10 Institute of Infectious Disease and Epidemiology, Tan Tock Seng Hospital, Singapore; 11 Division of Medicine, Christchurch Hospital, New Zealand; 12 Centre for Tropical Medicine, Nuffield Department of Clinical Medicine, University of Oxford, United Kingdom

**Keywords:** malaria, *Plasmodium falciparum*, *Plasmodium vivax*, *Plasmodium malariae*, thrombocytopenia, Indonesia, platelets

## Abstract

**Background:**

The significance of thrombocytopenia to the morbidity and mortality of malaria is poorly defined. We compared the platelet counts and clinical correlates of patients with and those without malaria in southern Papua, Indonesia.

**Methods:**

Data were collated on patients presenting to a referral hospital between April 2004 and December 2012.

**Results:**

Platelet measurements were available in 215 479 patients (23.4%), 66 421 (30.8%) of whom had clinical malaria. Patients with *Plasmodium falciparum* monoinfection had the lowest platelet counts and greatest risk of severe thrombocytopenia (platelet count, <50 000 platelets/µL), compared with those without malaria (adjusted odds ratio [OR], 6.03; 95% confidence interval [CI], 5.77–6.30]). The corresponding risks were 5.4 (95% CI, 5.02–5.80) for mixed infections, 3.73 (95% CI, 3.51–3.97) for *Plasmodium vivax* infection, and 2.16 (95% CI, 1.78–2.63) for *Plasmodium malariae* infection (*P* < .001). In total, 1.3% of patients (2701 of 215 479) died. Patients with severe malarial anemia alone (hemoglobin level, <5 g/dL) had an adjusted OR for death of 4.93 (95% CI, 3.79–6.42), those with severe malarial thrombocytopenia alone had an adjusted OR of 2.77 (95% CI, 2.20–3.48), and those with both risk factors had an adjusted OR of 13.76 (95% CI, 10.22–18.54; *P* < .001).

**Conclusions:**

Severe thrombocytopenia identifies both children and adults at increased risk of death from falciparum or vivax malaria, particularly in those with concurrent severe anemia.

Malaria remains an important threat to public health, particularly in communities with poor resources. Although *Plasmodium falciparum* accounts for the majority of severe malarial disease in sub-Saharan Africa, outside of Africa, nonfalciparum malarias are responsible for an increasing proportion of infections [1]. Anemia is a common manifestation of *Plasmodium* infection and is responsible for substantial morbidity, as well as for direct and indirect mortality [2, 3]. Thrombocytopenia is also a common feature of malaria due to all *Plasmodium* species [4–6], but in the absence of significant bleeding it is not regarded as a defining clinical manifestation of severe malaria [7]. In most cases, thrombocytopenia is not associated with bleeding and requires no treatment, with the platelet count rapidly returning to normal after successful treatment of the malarial episode. Pregnant women and infants appear to be at increased risk of thrombocytopenia, although the adverse consequences of this are unclear [8, 9]. Severe thrombocytopenia (defined as a platelet count of <50 000 platelets/μL) is reported in both *P. falciparum* and *Plasmodium vivax* infections, and although it has been associated with bleeding [10, 11] and disseminated intravascular coagulation [12–14], hemorrhagic manifestations are unusual. More recently, low platelet counts have been associated with mortality in patients with *P. falciparum* [15] and *P. vivax* infections [16, 17]. However, other studies have not demonstrated an association between thrombocytopenia and significant clinical risk [18, 19].

The present study is part of a prospective surveillance of clinical and laboratory data from Mitra Masyarakat Hospital in southern Papua, Indonesia, an area where 4 species of malaria are coendemic. This analysis was conducted to establish the comparative platelet counts of patients infected by the different *Plasmodium* species and to define the associated risks of morbidity and mortality.

## METHODS

### Study Site

Mimika district lies in south-central Papua, the easternmost province of Indonesia. Its geography, climate, and demographic characteristics have been described elsewhere [3, 20]. In brief, malaria transmission occurs throughout the year but is limited to the lowland areas. The average annual incidence of parasitemia is estimated to be 876 episodes per 1000 people, with most cases due to *P. falciparum*. The prevalence of asexual parasitemia in 2005 was estimated to be 7.5% for *P. falciparum,* 6.4% for *P. vivax,* 1.9% for mixed infection, and 0.6% for *Plasmodium malariae* [20]. Until November 2008, Rumah Sakit Mitra Masyarakat (RSMM) was the only referral hospital in the district, and since 2008 approximately 80% of patients with malaria attending an inpatient facility in the district have been treated there. RSMM has a capacity of 110 beds, with a high-dependency unit, a 24-hour emergency department, and an outpatient department that reviews approximately 300 patients per day, 6 days per week. The age distribution of all patient presentations peaks in infancy, with a second peak among individuals aged in their late 20s [3], whereas the absolute number of patient presentations with malaria peaks during the second year of life. Vivax malaria is the dominant cause of malaria in patients <3 years of age in both the outpatient and inpatient setting [9], and thereafter, *P. falciparum* is the most common malaria parasite [3, 20].

### Laboratory and Data Collection Procedures

Hospital protocols recommend that all patients presenting to the outpatient department with a fever and that all inpatients, regardless of diagnosis, should have a blood film performed for detection of malaria parasites. Microbiological diagnosis of malaria is based on a thick blood film examination, with confirmatory thin blood films and rapid diagnostic tests for *P. falciparum* also performed in some cases. Microscopy quality control of the hospital laboratory suggests >90% accuracy [21].

On their first presentation to RSMM, every patient is assigned a unique hospital record number, and this is used to link all clinical and laboratory data from all presentations. Demographic and administrative information is recorded by hospital clerks, along with the diagnosis from the attending physician (classified according to the International Classification of Diseases) and any deaths. For the purposes of analyses, ethnicity was categorized as Highland Papuan, Lowland Papuan, or non-Papuan, based on location of the clans' village(s). Complete blood counts are ordered according to clinical indication and are generated by coulter counter (JT Coulter, Ramsey, Minnesota).

### Data Merging and Statistical Analyses

**Figure 1.**
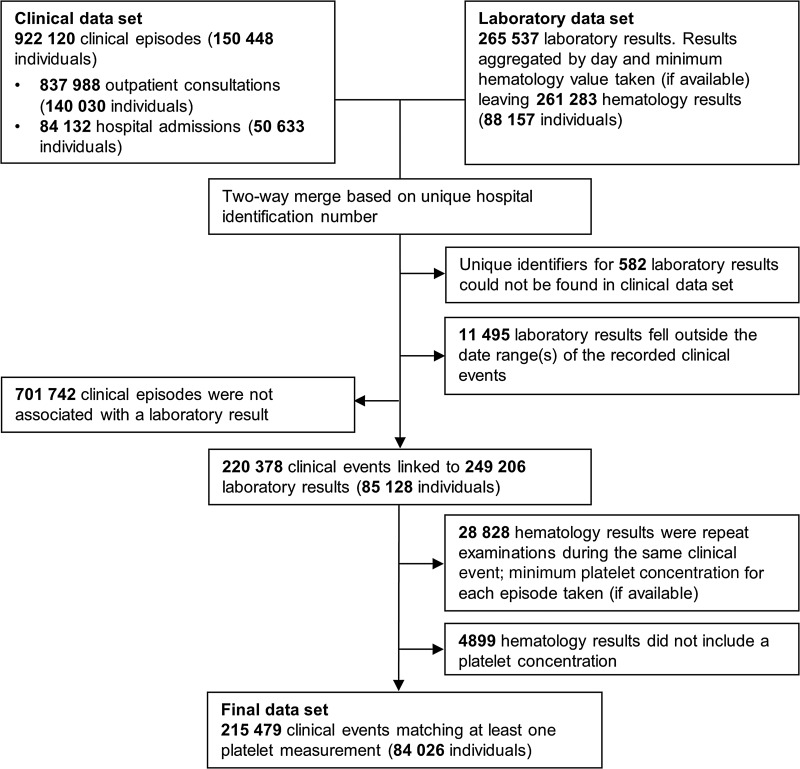
Flow diagram of the data merging process. Individual patients could present on multiple occasions and, in some cases, with multiple platelet measurements within each clinical episode. To highlight the degree of multiple sampling, the number of individuals who contributed to these presentations is provided in parentheses.

All statistical analyses were done in Stata, version 12.1. Clinical and hematology data were merged using the unique hospital record number and date of presentation. If >1 laboratory measurement was available for a single presentation, the minimum platelet count was taken (Figure 1). The primary outcome in this study was the mean number of platelets per microliter associated with infection for each *Plasmodium* species, compared with patients without malaria. Secondary measures included the risk of severe thrombocytopenia, the population attributable fraction (PAR) of severe thrombocytopenia associated with infection by the different *Plasmodium* species, and all-cause mortality. Thrombocytopenia was defined as severe if the platelet count was <50 000 platelets/μL (approximately the fifth percentile) and very severe if the count was <20 000 platelets/μL (approximately the first percentile; Supplementary Figure 1). Severe anemia was defined as a hemoglobin concentration of <5 g/dL. Continuous data were analyzed using linear regression, and binary data (such as severe thrombocytopenia and death) were analyzed using logistic regression. Since some patients appeared in the database multiple times, robust standard errors were calculated using the clustered sandwich estimator.

For the purposes of these analyses, mixed infections were defined as concomitant infection with any combination of *Plasmodium* species. Univariable and multivariable analyses were performed for each of the following variables: infecting *Plasmodium* species (negative, *P. falciparum, P. vivax, P. malariae, P. ovale*, or mixed species), sex, ethnicity (non-Papuan, Highland Papuan, or Lowland Papuan), age group (<1 year, 1 to <5 years, 5 to <15 years, and ≥15 years), year of presentation (2004–2012), department (outpatient vs inpatient), and number of presentations with malaria in the preceding 2 months. Fractional polynomials were used to define the nonlinear relationship between age and the mean platelet count and risk of severe thrombocytopenia [22], but to maintain the stability of these models the following patients were excluded: patients with platelet counts of <5000 or >1 000 000 platelets/μL (338 [0.16%]), infants <1 week of age (1073 [0.50%]), and adults >63 years of age (the 99th percentile; 2205 [1.02%]).

Adjusted PAFs of severe thrombocytopenia were calculated from multivariable logistic regression models, using the punaf module for Stata, which derives PAFs by means of the formulae provided in Greenland and Dreschler [23]. Because of very small numbers, data from patients with *P. ovale* infections (30 [0.01%]) are included in the baseline values and the univariable analyses but excluded from the multivariable analyses.

### Ethics Approval

Ethics approval for this study was obtained from the Health Research Ethics Committees of the University of Gadjah Mada, Indonesia, and the Menzies School of Health Research, Darwin, Australia. Since data were gathered from routine hospital surveillance, informed consent was not requested from participants. However, all records were anonymized, to ensure patient confidentiality.

## RESULTS

Of the 922 120 patient presentations to the Mitra Masyarakat Hospital between April 2004 and December 2012, 837 989 (90.9%) were to the outpatient department alone, and 84 131 (9.1%) resulted in hospital admission (Table 1). Microscopically confirmed malaria was diagnosed in 18.3% of patient presentations (168 525), with *P. falciparum* accounting for 53.3% of monoinfections, *P. vivax* for 32.3%, *P. malariae* for 2.7%, and *P. ovale* for 0.07%. Mixed-species infections were detected in 19 569 presentations (11.6%), which, in 18 489 (94.5%) cases, were mixed *P. falciparum* and *P. vivax* infections (Table 1).

**Table 1.** Distribution of Clinical and Laboratory Data and Thrombocytopenia Status, by Clinical and Demographic Group

Variable	Clinical Events, No.	Patients, No.	Distribution of Clinical and Laboratory Data				Platelet Data			
			OP Events, No.	OP Events With Platelet Count, No. (%)^a^	IP Events, No.	IP Events With Platelet Count, No. (%)^a^	Mean Platelet Count (per 1000)	*P* Value^b^	< 50 000 Platelets/μL, No. (%)	*P* Value^b^
Infecting *Plasmodium* species										
None (negative test results)	753 595	135 229	695 051	105 432 (15.2)	58 544	43 626 (74.5)	256.37 ± 141.38	Ref	4084 (2.7)	Ref
*P. falciparum*	89 748	44 171	73 392	21 828 (29.7)	16 356	14 931 (91.3)	129.03 ± 100.47	<.001	5722 (15.6)	<.001
*P. vivax*	54 495	28 841	49 041	14 512 (29.6)	5454	5040 (92.4)	165.42 ± 121.37	<.001	1650 (8.4)	<.001
>1 (mixed infection)	19 569	14 206	16 210	5411 (33.4)	3359	3089 (92)	128.98 ± 96.19	<.001	1148 (13.5)	.203
*P. malariae*	4598	4045	4190	1198 (28.6)	408	382 (93.6)	142.66 ± 98.15	<.001	116 (7.3)	<.001
*P. ovale*	115	110	105	22 (21)	10	8 (80)	146.2 ± 79.97	<.001	2 (6.7)	<.001
Sex										
Female	506 055	70 493	458 599	78 552 (17.1)	47 456	38 562 (81.3)	219.24 ± 136.35	Ref	6051 (5.2)	Ref
Male	416 065	80 186	379 390	69 851 (18.4)	36 675	28 514 (77.7)	222.03 ± 149.37	.008	6671 (6.8)	<.001
Ethnic group										
Non-Papuan	149 017	44 196	136 604	22 851 (16.7)	12 413	9146 (73.7)	247.13 ± 123.31	Ref	822 (2.6)	Ref
Highland Papuan	644 073	79 443	586 388	105 757 (18)	57 685	47 318 (82)	203.73 ± 137.81	<.001	11 235 (7.3)	<.001
Lowland Papuan	127 385	26 413	113 440	19 589 (17.3)	13 945	10 555 (75.7)	277.5 ± 164.07	<.001	654 (2.2)	.003
Age, y										
<1	70 221	20 086	55 359	12 998 (23.5)	14 862	6595 (44.4)	323.49 ± 177.22	<.001	651 (3.3)	<.001
1 to <5	139 033	23 596	124 985	29 337 (23.5)	14 048	11 230 (79.9)	279.93 ± 170.61	<.001	1403 (3.5)	<.001
5 to <15	107 163	23 318	100 319	19 341 (19.3)	6844	6177 (90.3)	214.15 ± 136.73	<.001	1478 (5.8)	<.001
≥15	605 588	101 728	557 250	86 659 (15.6)	48 338	43 046 (89.1)	187.57 ± 111.13	Ref	9188 (7.1)	Ref
Malaria presentations in the past 2 mo, no.										
0	799 988	150 448	724 429	126 653 (17.5)	75 559	59 237 (78.4)	221.41 ± 142.98	Ref	11 268 (6.1)	Ref
≥1	122 132	31 080	113 560	21 750 (19.2)	8572	7839 (91.4)	214.90 ± 138.89	<.001	1454 (4.9)	<.001
Year										
2004	62 985	25 864	56 227	8316 (14.8)	6758	4495 (66.5)	205.66 ± 149.48	Ref	1045 (8.2)	Ref
2005	88 400	30 950	78 398	16 676 (21.3)	10 002	8498 (85)	219.91 ± 153.23	<.001	1688 (6.7)	<.001
2006	96 086	34 260	86 288	12 667 (14.7)	9798	8312 (84.8)	243.51 ± 160.02	<.001	1.025 (4.9)	<.001
2007	106 046	36 914	95 272	12 872 (13.5)	10 774	8469 (78.6)	220.4 ± 148.08	<.001	1533 (7.2)	.002
2008	98 074	34 127	88 967	11 184 (12.6)	9107	7504 (82.4)	211.83 ± 134.54	.001	1245 (6.7)	<.001
2009	110 796	34 341	100 756	20 165 (20)	10 040	8188 (81.6)	212.5 ± 133.49	<.001	1716 (6.1)	<.001
2010	112 566	35 041	103 263	22 152 (21.5)	9303	7525 (80.9)	214.67 ± 136.52	<.001	1735 (5.8)	<.001
2011	115 683	37 055	106 640	18 840 (17.7)	9043	6654 (73.6)	225.24 ± 133.65	<.001	1230 (4.8)	<.001
2012	131 484	41 495	122 178	25 531 (20.9)	9306	7431 (79.9)	225.64 ± 136.95	<.001	1505 (4.6)	<.001
Overall	922 120	150 448	837 989	148 403 (17.7)	84 131	67 076 (79.7)	220.52 ± 142.44		12 722 (5.9)	

Abbreviations: IP, inpatient; OP, outpatient; Ref, reference.

a Events in which the platelet count was measured.

b Based on univariable linear regression with correction of the variance-covariance matrix for within-patient correlation.

### Availability of Platelet Data

Overall, 215 479 presentations (23.4%) could be matched with at least 1 platelet count measurement (Figure 1). A greater proportion of outpatient visits were linked to a platelet count measurement if the patient had malaria rather than no malaria (30.1% vs 15.2%, respectively; *P* < .001). The corresponding figures for patients admitted to the wards were (91.6% vs 74.5%; *P* < .001). Patients who had a platelet measurement had a median age of 21.0 years, compared with 23.0 years among those without a measurement. Measurement of platelets was also slightly more common in males, compared with females (23.6% vs 23.1%), and in Papuans, compared with non-Papuans (23.7% vs 21.5%).

### Reduction in Platelet Concentration Associated With Malaria

The mean platelet count was significantly lower in Highland Papuans (204 × 10^3^ platelets/μL), compared with that for Lowland Papuans (278 × 10^3^ platelets/μL) and non-Papuans (247 × 10^3^ platelets/μL; *P* < .001 for both comparisons). Whereas the overall risk of severe thrombocytopenia with each presentation fell to <2% from early childhood in non-Papuans and lowlanders, highlanders remained at significantly greater risk throughout adulthood (*P* < .001; Supplementary Figure 2).

**Figure 2.**
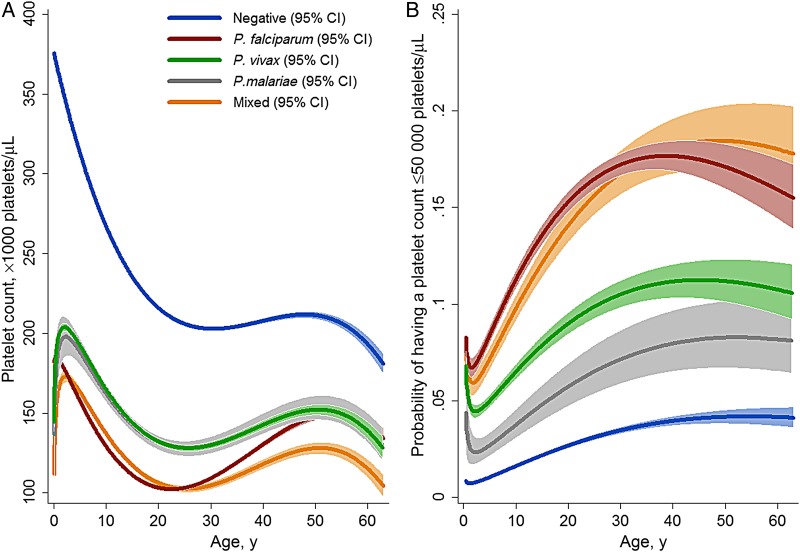
Estimated mean platelet count among hospital attendees (*A*) and estimated probability of severe thrombocytopenia (platelet concentration, <50 000 platelets/µL; *B*), by *Plasmodium* species. Figures were generated by multiple fractional polynomial regression analyses with the following covariables: *Plasmodium* species, by age, sex, ethnic group, and year. Bands represent 95% CIs. Abbreviation: CI, confidence interval.

The mean platelet concentration and prevalence of severe thrombocytopenia varied significantly with *Plasmodium* species (Table 1). Overall, *P. falciparum,* alone or as part of a mixed infection, was associated with the greatest difference in mean platelet counts, compared with counts for individuals without malaria (−127 × 10^3^ platelets/μL [95% confidence interval [CI], −126 to −129 × 10^3^ platelets/μL]; *P* < .001). After adjustment for confounding factors, patients presenting to hospital with malaria had lower mean platelet counts and higher odds of severe thrombocytopenia than patients without malaria at all ages (Figure 2). Patients with *P. falciparum* (alone or as part of mixed infections) without a history of presentation to the hospital with malaria within the preceding 2 months had significantly lower platelet counts (127 × 10^3^ platelets/μL [95% CI, 126 × 10^3^–128 × 10^3^ platelets/μL]), compared with those with a single recent episode of malaria (140 × 10^3^ platelets/μL [95% CI, 138 × 10^3^–143 × 10^3^ platelets/μL]) and those with ≥2 episodes (149 × 10^3^ platelets/μL [95% CI, 143 to 155 × 10^3^ platelets/μL]). The influence of recent history of malaria was not apparent in patients presenting without malaria or in those with *P. vivax* monoinfection.

### Risk of Severe Thrombocytopenia

In total, 75 029 (34.8%) of the 215 479 presentations were associated with a platelet count of <150 × 10^3^ platelets/μL, 12 722 (5.9%) had counts of <50 × 10^3^ platelets/μL, and 2001 (0.9%) had counts of <20 × 10^3^ platelets/μL. Compared with patients without malaria, patients with *P. falciparum* infection were at the greatest risk of severe thrombocytopenia (adjusted odds ratio [OR], 6.03 [95% CI, 5.77–6.30]), followed by those with mixed infections (adjusted OR, 5.40 [95% CI, 5.02–5.80]), those with *P. vivax* infection (adjusted OR, 3.73 [95% CI, 3.51–3.97]), and those with *P. malariae* infection (adjusted OR, 2.16 [95% CI, 1.78–2.63]); *P* < .001 for all comparisons; Table 2).

**Table 2.** Risk Factors for Severe Thrombocytopenia

Risk Factor	Univariable Analysis		Multivariable Analysis		
	Crude OR (95% CI)	*P* Value	Adjusted OR (95% CI)	*P* Value	Overall PAF, % (95% CI)
Infecting *Plasmodium* species					
None (negative test results)	Reference		Reference		
*P. falciparum*	6.54 (6.28–6.83)	<.001	6.03 (5.77–6.30)	<.001	35.88 (34.92–36.83)
*P. vivax*	3.27 (3.08–3.47)	<.001	3.73 (3.51–3.97)	<.001	9.14 (8.55–9.72)
*P. malariae*	2.81 (2.32–3.41)	<.001	2.16 (1.78–2.63)	<.001	0.47 (.31–0.62)
>1 (mixed infection)	5.54 (5.17–5.94)	<.001	5.40 (5.02–5.80)	<.001	7.04 (6.58–7.51)
Age, y					
≥15	Reference		Reference		
<1	0.45 (.42–.49)	<.001	0.47 (.43–.51)	<.001	<0
1 to <5	0.47 (.44–.50)	<.001	0.32 (.30–.34)	<.001	<0
5 to <15	0.81 (.76–.85)	<.001	0.50 (.47–.53)	<.001	<0
Sex					
Female	Reference		Reference		
Male	1.34 (1.29–1.38)	<.001	1.39 (1.34–1.44)	<.001	12.99 (11.49–14.46)
Ethnicity					
Non-Papuan	Reference		Reference		
Highland Papuan	3.00 (2.80–3.23)	<.001	2.75 (2.56–2.97)	<.001	52.71 (49.92–55.34)
Lowland Papuan	0.84 (.76–.93)	.001	0.96 (.86–1.07)	.430	<0
Hb level <5 g/dL^a^					
No	Reference		Reference		
Yes	4.02 (3.79–4.27)	<.001	3.16 (2.96–3.37)	<.001	7.00 (6.49–7.51)
Year^b^					
2004	Reference		Reference		…
2005	0.81 (.75–.88)	<.001	0.86 (.79–.94)	<.001	…
2006	0.58 (.53–.63)	<.001	0.65 (.59–.71)	<.001	…
2007	0.87 (.80–.95)	.001	0.98 (.90–1.07)	.590	…
2008	0.80 (.74–.88)	<.001	1.08 (.98–1.18)	.110	…
2009	0.73 (.67–.79)	<.001	0.97 (.89–1.06)	.516	…
2010	0.70 (.65–.76)	<.001	0.96 (.88–1.05)	.375	…
2011	0.57 (.52–.62)	<.001	0.83 (.76–.91)	<.001	…
2012	0.54 (.50–.58)	<.001	0.72 (.66–.79)	<.001	…
Department^c^					
Outpatient	Reference		Excluded		…
Inpatient	2.38 (2.30–2.47)	<.001	…		…
Malaria presentations in the past 2 mo, no.					
0	Reference		Reference		…
≥1	0.80 (.76–.85)	<.001	0.71 (.67–0.75)	<.001	<0

Severe thrombocytopenia was defined as a platelet count of <50 000 platelets/μL.

Abbreviations: CI, confidence interval; Hb, hemoglobin; OR, odds ratio; PAF, population attributable fraction.

a Criterion for anemia.

b Included in the model for calculation of overall PAFs, but PAFs are not presented.

c Not included in the multivariable model because admission was deemed to be a consequence of severe disease, rather than a confounder.

Patients with a recent presentation to the hospital with malaria due to any *Plasmodium* species were at a lower risk of severe thrombocytopenia, compared with those with no recent malaria (adjusted OR, 0.71 [95% CI, .67–.75]; *P* < .001; Table 2). The effect of recent malaria was most noticeable in patients presenting with *P. falciparum* infection (adjusted OR, 0.50 [95% CI, .45–.55]; *P* < .001), compared with those without malaria (adjusted OR, 0.89 [95% CI, .80–.98]; *P* = .017) or those with *P. vivax* (adjusted OR, 0.96 [95% CI, .84–1.09]; *P* = .510).

In total, 215 044 (99.8%) of patients with platelet counts available also had hemoglobin levels measured; of these, 3.7% (7931) had severe anemia. The risk of severe thrombocytopenia was 18.7% (1484 of 7931) among patients with severe anemia, compared with 5.4% (11 221 of 207 113) among those without severe anemia (adjusted OR, 3.16 [95% CI, 2.96–3.37]; *P* < .001). The overall adjusted population fraction of severe thrombocytopenia attributable to *P. falciparum* infection was 35.9% (95% CI, 34.9%–36.8%), with 9.1% (95% CI, 8.6%–9.7%) attributable to *P. vivax,* 0.5% (95% CI, 0.3%–0.6%) attributable to *P. malariae*, and 7.0% (95% CI, 6.6%–7.5%) attributable to mixed species infections (Table 2).

### Consequences of Severe Thrombocytopenia

**Figure 3.**
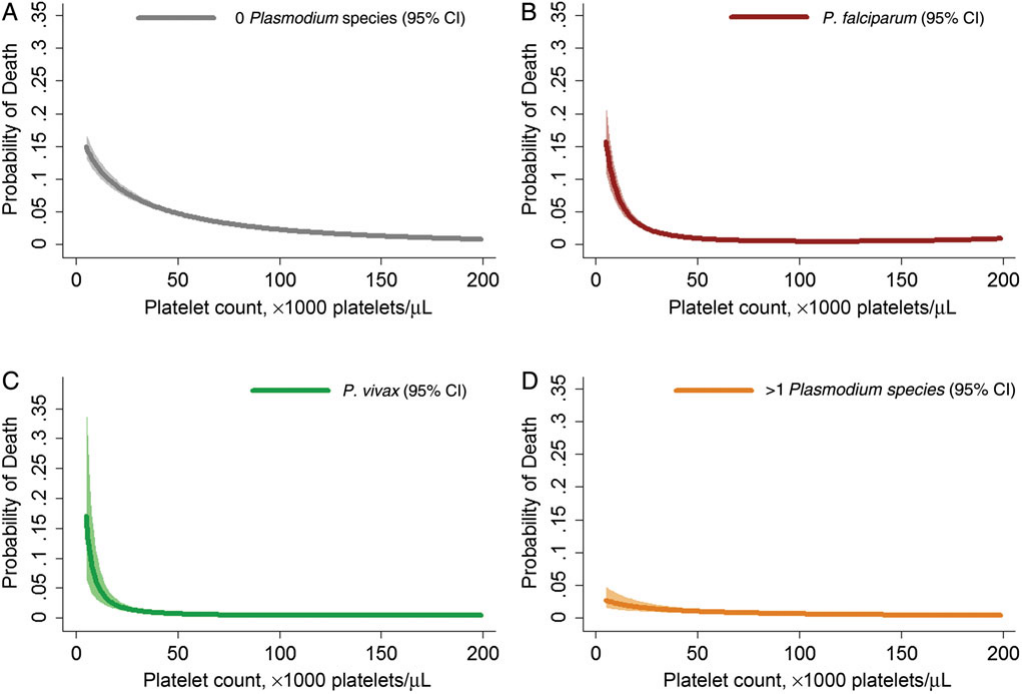
Estimated probability of mortality, by platelet count. Figures were generated by multiple fractional polynomial regression analyses (with adjustment for age, sex, ethnic group, and year), according to infecting *Plasmodium* species, as follows: 0 *Plasmodium* species (negative test results; *A*), *P. falciparum* (*B*), *P. vivax* (*C*), and >1 *Plasmodium* species (mixed infection; *D*). Abbreviation: CI, confidence interval.

Admission for inpatient care was required in 49.6% of patients (6196 12 499) initially presenting to the outpatient department with severe thrombocytopenia, compared with 29.0% (57 966 of 200 066) among patients without severe thrombocytopenia (OR, 2.41 [95% CI, 2.32–2.50]; *P* < .001). Overall, 1.3% of patients (2701 of 215 479) with a platelet measurement died, with the risk of death rising exponentially as the platelet count fell (Figure 3). The mortality risk among patients with severe thrombocytopenia (<50 000 platelets/μL) was 7.9% (324 of 4084) among those without malaria, 2.1% (120 of 5722) among those with *P. falciparum* infection, 1.5% (25 of 1650) among those with *P. vivax* infection, 1.7% (2 of 114) among those with *P. malariae* infection, and 1.7% (19 of 1148) among those with mixed infections. When platelet counts fell to <20 000 platelets/μL, the risk of death increased to 11% (108 of 978) among patients without malaria, 5.6% (40 of 708) among those with *P. falciparum* infection, 3.6% (6 of 168) among those with *P. vivax* infection, 0% (0 of 15) among those with *P. malariae* infection, and 3.1% (4 of 131) among those with mixed infections.

Overall, compared with patients with neither severe anemia nor thrombocytopenia, the adjusted ORs for death were 5.21 (95% CI, 4.53–5.98) among those with severe anemia alone, 4.65 (95% CI, 4.10–5.28) among those with severe thrombocytopenia alone, and 16.44 (95% CI, 13.70–19.74) among those with both (Table 3). This relationship was apparent in both children and adults with malaria and in *P. falciparum*, *P. vivax*, and mixed infections (Table 4).

**Table 3.** Univariable and Multivariable Analyses of the Risk Factors for Death Among 215 449 Patients

Variable	Univariable Analysis		Multivariable Analysis		
	Crude OR (95% CI)	*P* Value	Adjusted OR (95% CI)	*P* Value	PAF, % (95% CI)
Platelet count, Hb level^a^					
≥50 000 platelets/μL					
≥5 g/dL	Reference		Reference		…
<5 g/dL	3.92 (3.43–4.49)	<.001	5.21 (4.53–5.98)	<.001	7.26 (6.17–8.33)
<50 000 platelets/μL					
≥5 g/dL	3.07 (2.73–3.45)	<.001	4.65 (4.10–5.28)	<.001	9.71 (8.43–10.97)
<5 g/dL	11.28 (9.49–13.42)	<.001	16.44 (13.70–19.74)	<.001	5.24 (4.42–6.04)
Infecting *Plasmodium* species					
None (negative test results)	Reference		Reference		
*P. falciparum*	0.61 (.54–0.69)	<.001	0.43 (.38–.49)	<.001	<0
*P. vivax*	0.38 (.31–.46)	<.001	0.32 (.26–.39)	<.001	<0
*P. malariae*	0.56 (.32–.96)	.036	0.50 (.29–.87)	.014	<0
>1 (mixed infection)	0.49 (.38–.63)	<.001	0.36 (.28–.47)	<.001	<0
Age, y					
≥15	Reference		Reference		…
<1	1.37 (1.22–1.53)	<.001	1.47 (1.30–1.65)	<.001	4.24 (2.78–5.67)
1 to <5	0.60 (.53–.67)	<.001	0.72 (.64–.82)	<.001	<0
5 to <15	0.56 (.48–.65)	<.001	0.65 (.56–.76)	<.001	<0
Sex					
Female	Reference		Reference		…
Male	1.58 (1.46–1.71)	<.001	1.63 (1.51–1.77)	<.001	21.56 (18.10–24.87)
Ethnicity					
Non-Papuan	Reference		Reference		…
Highland Papuan	0.61 (.56–.67)	<.001	0.60 (.54–.66)	<.001	<0
Lowland Papuan	0.93 (.83–1.05)	.267	0.96 (.85–1.09)	.540	<0
Year^b^					
2004	Reference		Reference		…
2005	0.98 (.80–1.20)	.867	1.11 (.91–1.36)	.316	…
2006	1.41 (1.16–1.71)	.001	1.55 (1.27–1.89)	<.001	…
2007	1.57 (1.29–1.90)	<.001	1.55 (1.28–1.89)	<.001	…
2008	1.84 (1.52–2.22)	<.001	1.82 (1.50–2.21)	<.001	…
2009	0.92 (.75–1.12)	.401	1.04 (.85–1.27)	.687	…
2010	0.76 (.62–0.93)	.009	0.87 (.71–1.07)	.201	…
2011	0.93 (.76–1.14)	.497	1.07 (.87–1.31)	.513	…
2012	0.87 (.71–1.06)	.158	1.03 (.85–1.26)	.748	…
Malaria presentations in the past 2 mo, no.					
0	Reference		Reference		…
≥1	0.63 (.55–.72)	<.001	0.80 (.69–.91)	.001	<0

Abbreviations: CI, confidence interval; Hb, hemoglobin; OR, odds ratio; PAF, population attributable fraction.

a Severe thrombocytopenia was defined as a platelet count of <50 000 platelets/μL, and anemia was defined as a hemoglobin (Hb) level of <5 g/dL.

b Included in the model for calculation of overall PAFs, but PAFs are not presented.

**Table 4.** Risk of Mortality According to Severe Thrombocytopenia, Severe Anemia, and Malaria Status

Variable	Platelet Count ≥50 000 Platelets/μL, Hb Level <5 g/dL		Platelet Count <50 000 Platelets/µL, Hb Level ≥5 g/dL		Platelet Count <50 000 Platelets/µL, Hb Level <5 g/dL	
	Adjusted OR (95% CI)^a^	*P* Value	Adjusted OR (95% CI)^a^	*P* Value	Adjusted OR (95% CI)^a^	*P* Value
Infecting *Plasmodium* species^a^						
None (negative test results)	5.10 (4.32–6.01)	<.001	6.21 (5.37–7.20)	<.001	17.14 (13.61–21.58)	<.001
*P. falciparum*	5.05 (3.64–7.00)	<.001	2.71 (2.06–3.55)	<.001	16.97 (11.81–24.38)	<.001
*P. vivax*	4.04 (2.22–7.33)	<.001	2.67 (1.55–4.60)	<.001	9.21 (4.53–18.73)	<.001
>1 (mixed infection)	5.29 (2.69–10.43)	<.001	2.52 (1.26–5.05)	.009	9.77 (4.39–21.73)	<.001
Any, overall	4.93 (3.79–6.42)	<.001	2.77 (2.20–3.48)	<.001	13.76 (10.22–18.54)	<.001
Age among malarial patients, y^b^						
<1	4.36 (1.80–10.56)	.001	3.96 (1.55–10.09)	.004	7.17 (2.60–19.79)	<.001
1 to <5	3.23 (1.92–5.43)	<.001	1.77 (.84–3.71)	.133	11.89 (6.52–21.66)	<.001
5 to <15	6.35 (3.24–12.46)	<.001	2.40 (1.14–5.04)	.021	5.98 (1.41–25.40)	.015
≥15	6.18 (4.28–8.92)	<.001	2.87 (2.19–3.77)	<.001	20.05 (13.57–29.62)	<.001

Abbreviations: CI, confidence interval; Hb, hemoglobin; OR, odds ratio.

a Model presents risk with respect to the reference group of patients without severe anemia or thrombocytopenia (severe thrombocytopenia was defined as a platelet count of <50 000 platelets/μL, and anemia was defined as a Hb level of <5 g/dL). Adjusted for age group, sex, ethnicity, year, and recent malaria presentations in the past 2 months.

b Model presents risk in patients with malaria, with respect to the reference group of patients without severe anemia or thrombocytopenia (severe thrombocytopenia was defined as a platelet count of <50 000 platelets/μL, and anemia was defined as a Hb level of <5 g/dL). Adjusted for species, sex, ethnicity, year, and recent malaria presentations in the past 2 months.

In the absence of severe anemia, the greatest risk of mortality associated with severe thrombocytopenia was among patients without malaria (adjusted OR, 6.21 [95% CI, 5.37–7.20]; *P* < .001), whereas the risk among patients with malaria was 2.77 (95% CI, 2.20 to 3.480; *P* < .001), with no difference between infecting species (Table 4). The overall PAF of death associated with severe thrombocytopenia was 14.6% (95% CI, 13.1%–16.0%); the full multivariable model for mortality is presented in Table 3. There was no significant difference in the risk of bleeding recorded in patients with (4.3% [21 of 490]) and those without (5.7% [126 of 2211]) severe thrombocytopenia (*P* = .228).

## DISCUSSION

In this very large hospital-based surveillance study, almost two thirds of patients with acute malaria had thrombocytopenia (platelet count, <150 000 platelets/μL), with 13% of patients presenting with platelet counts of <50 000 platelets/μL. The greatest risk of severe thrombocytopenia was in patients infected with *P. falciparum*, either alone or mixed (OR, 5.4–6.1), accounting for >40% of observed cases. Severe thrombocytopenia was associated with a 2.4-fold greater risk of admission to hospital and a 4.7-fold increased risk of death, rising to 16-fold when both severe anemia and severe thrombocytopenia were present (Table 3). Similar relationships between the risk of death and severe thrombocytopenia were seen in both children and adults with malaria and in cases of *P. falciparum* and *P. vivax* infections.

Malaria causes a variety of hematological insults arising from hemolysis, host inflammatory response, hematopoietic suppression, and splenic pooling [24, 25]. Severe anemia is an important prognostic indicator of fatal outcome, particularly in young children [3, 26]. While thrombocytopenia is also extremely common, its contribution to morbidity and mortality has been less clear. In patients with falciparum malaria, severe disease and mortality are increased with severe thrombocytopenia [15, 27], and more recently this has also been observed in patients with severe vivax malaria [16]. Many previous studies examining predictors of malaria mortality have not included platelet counts [7]. Other studies showing no relationship between malarial thrombocytopenia and mortality have been smaller and may have been underpowered [28, 29].

Previous studies have shown a consistent inverse correlation between parasitemia at presentation and the platelet count [17], but our study did not record the peripheral parasite count routinely, and hence we were unable to explore this. In Papua, we have shown that peripheral parasitemia is considerably higher in symptomatic patients with *P. falciparum* infections, compared with patients infected with *P. vivax* [20, 30]. This may have contributed to the greater risk of severe thrombocytopenia in patients with P*. falciparum* infection (OR, 6.1), compared with those with *P. vivax* (OR, 3.7).

We have shown previously that the risk of anemia in this population is greatest in young patients, highlanders, and those presenting with recurrent episodes of malaria [3]. In contrast, in the current analysis, the risk of severe thrombocytopenia was significantly lower in patients with malaria who had had a prior episode of malaria within the preceding 2 months (OR, 0.8); this attenuation was most apparent in patients presenting with *P. falciparum* but not in those presenting with *P. vivax* monoinfection or without malaria. Furthermore, after the first year of life, lowland and non-Papuan patients had a low risk of severe thrombocytopenia. The risk of thrombocytopenia was significantly higher in Highland Papuans, and this was sustained throughout adulthood. Highlanders constitute an ethnic group originating from non–malaria-endemic regions who have not been under genetic selection pressure from malaria parasite infections. In the last decade, many highlanders have migrated at all ages to the lowland areas, where they have been exposed to malaria, often getting their first episodes of malaria in later life. Our findings are consistent with lowland ethnicity or recent malaria resulting in a reduction of the host inflammatory response to acute malaria and decreased platelet activation and consumption.

The pathogenic mechanisms by which platelets mediate disease severity remain to be delineated. However, clinical, autopsy, ex vivo, and in vitro studies have shown that platelets are involved in parasite sequestration [31], as well as in clumping and/or agglutination of infected and uninfected erythrocytes [32, 33]. Platelets express Toll-like receptors (TLRs), which, on recognition of *P. falciparum* molecular patterns, release prepackaged inflammatory mediators [34]. This could partially explain the attenuation with repeat exposure, as repeated stimulation of TLRs leads to decreased signaling and decreased inflammatory responses [35]. Nitric oxide (NO) is also a key mediator of platelet homeostasis, and the decreased NO bioavailability found in both children [36] and adults [37] with severe and fatal malaria may contribute to increased platelet activation and consumption.

Our large-scale observational study has a number of limitations. First, platelet counts were only available in 26% of all presentations, and so there may be a degree of residual confounding in our multivariable analyses. However, the available hematology data rose to 80% in patients requiring admission. Although the risk of thrombocytopenia was greater in inpatients than outpatients, the magnitude of the other risk factors remained similar in both departments. Second, the surveillance program did not document the presence of all severe manifestations of malaria in these patients, so it is not possible in this data set to determine whether the presence of severe thrombocytopenia would have identified patients at risk of death in whom other World Health Organization (WHO) criteria for severe disease were not apparent [7]. Previous studies have used multivariate analysis to identify biomarkers predictive of poor outcome, but most have not included platelet counts [7]. Hence, it is possible that the mortality risk associated with severe thrombocytopenia may be better represented by other clinical biochemical and inflammatory markers. However, platelet counts are readily available from an automated blood count, a routine laboratory test that is widely accessible even in referral inpatient facilities and even some more remote health posts and that is more accessible than other recognized laboratory predictors of mortality in WHO severity criteria (such lactate and bicarbonate levels or creatinine level) [7]. Our study, the largest to date that examined relationships between severe thrombocytopenia and malaria mortality, highlights that severe thrombocytopenia should serve as a warning sign of poor outcome, particularly when coexisting with severe anemia. We believe that severe thrombocytopenia may be useful in guiding the need for referral or triage to a ward where a higher level of care is provided. Our analysis focused on applying a threshold of 50 000 platelets/μL, which was associated with an overall mortality of 3.9%, a PAF of 14.5% and sufficient power to determine other relevant confounding factors. However, the mortality risk rose to 7.9% in patients with a platelet count of <20 000 platelets/μL (5.6% in falciparum malaria and 3.6% in vivax malaria). We propose that a platelet count of ≤20 000 platelets/μL should be included as a defining severity criterion for both falciparum and vivax malaria. Prospective studies are warranted to evaluate the prognostic value of using platelet counts in conjunction with hemoglobin concentrations to define medical interventions and to determine the underlying processes by which thrombocytopenia contributes to the pathology of malaria.

## Supplementary Data

Supplementary materials are available at *The Journal of Infectious Diseases* online (http://jid.oxfordjournals.org). Supplementary materials consist of data provided by the author that are published to benefit the reader. The posted materials are not copyedited. The contents of all supplementary data are the sole responsibility of the authors. Questions or messages regarding errors should be addressed to the author.

Supplementary Data
