# Supplementary material for: Severe Malarial Thrombocytopenia: A Risk Factor for Mortality in Papua, Indonesia
Source: J Infect Dis. 2014 Aug 28;211(4):623–34. doi: 10.1093/infdis/jiu487 (PMC4305266; doi:10.1093/infdis/jiu487)
Supplement: Supplementary Data [file supp_211_4_623__index.html]

Severe Malarial Thrombocytopenia: A Risk Factor for Mortality in Papua, Indonesia — Severe Malarial Thrombocytopenia: A Risk Factor for Mortality in Papua, Indonesia — Supplementary Data 

# Severe Malarial Thrombocytopenia: A Risk Factor for Mortality in Papua, Indonesia

## Supplementary Data

Supplementary Data

**Files in this Data Supplement:**

- Supplementary Data - Docx file
