# Supplementary material for: Severe Malarial Thrombocytopenia: A Risk Factor for Mortality in Papua, Indonesia
Source: J Infect Dis. 2014 Aug 28;211(4):623–34. doi: 10.1093/infdis/jiu487 (PMC4305266; doi:10.1093/infdis/jiu487)

**Supplementary Figure 1**. Histogram of platelet counts in patients with and without malaria.

Footnote: Vertical lines represent the 1^st^ (20,000 /µl) and 5^th^ (45,000 /µl) percentiles (20,000 and 45,000 /µl respectively).

**Supplementary Figure 2.** Estimated mean platelet count in hospital attendees by ethnicity (A) and risk of severe thrombocytopenia (platelet count less than 50,000 µl^-1^) (B).

Footnote: Figures generated by multiple fractional polynomial regression analyses with the following covariables: ethnic group by age, *Plasmodium* species, sex, and year. Bands represent 95% confidence intervals.


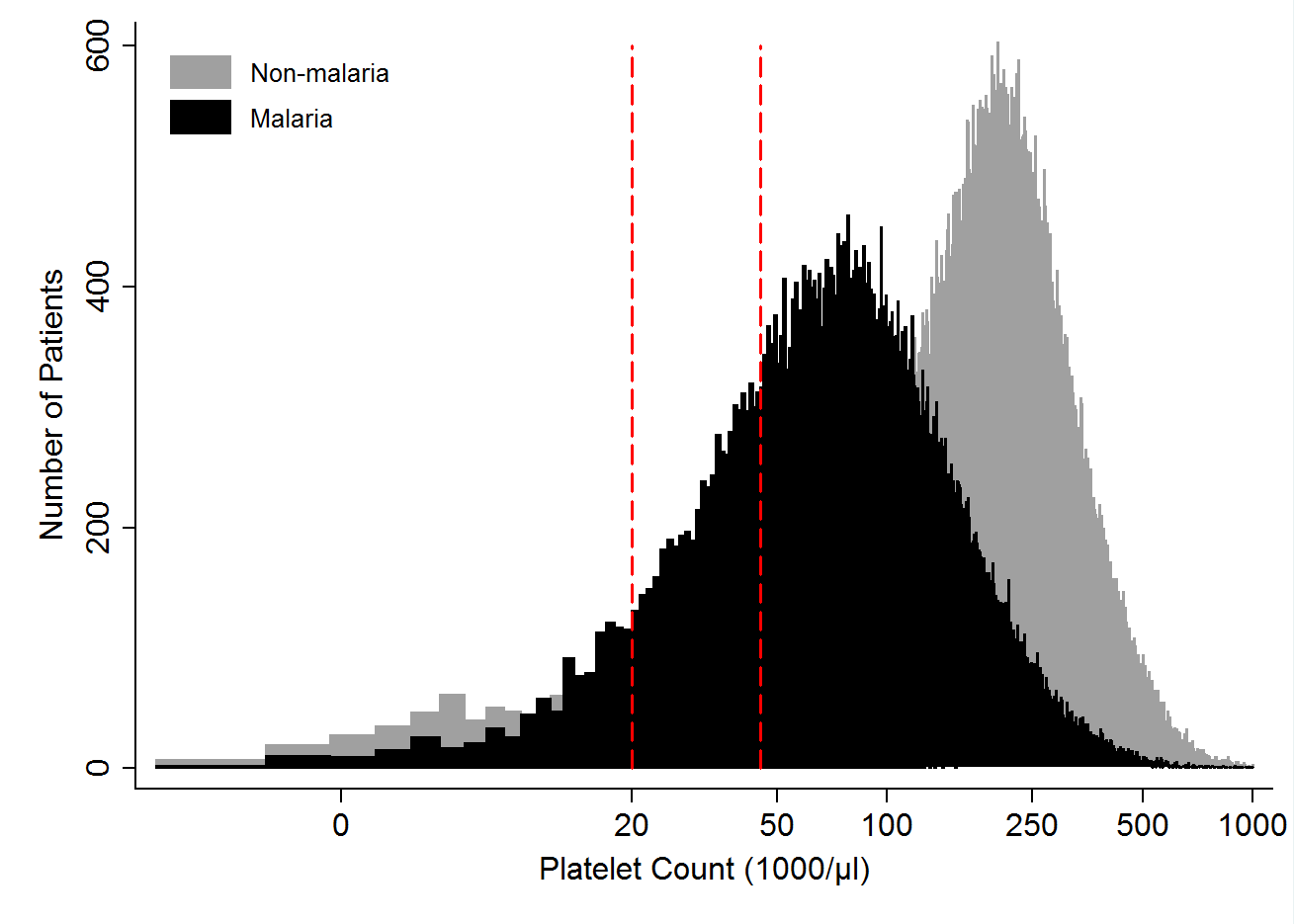


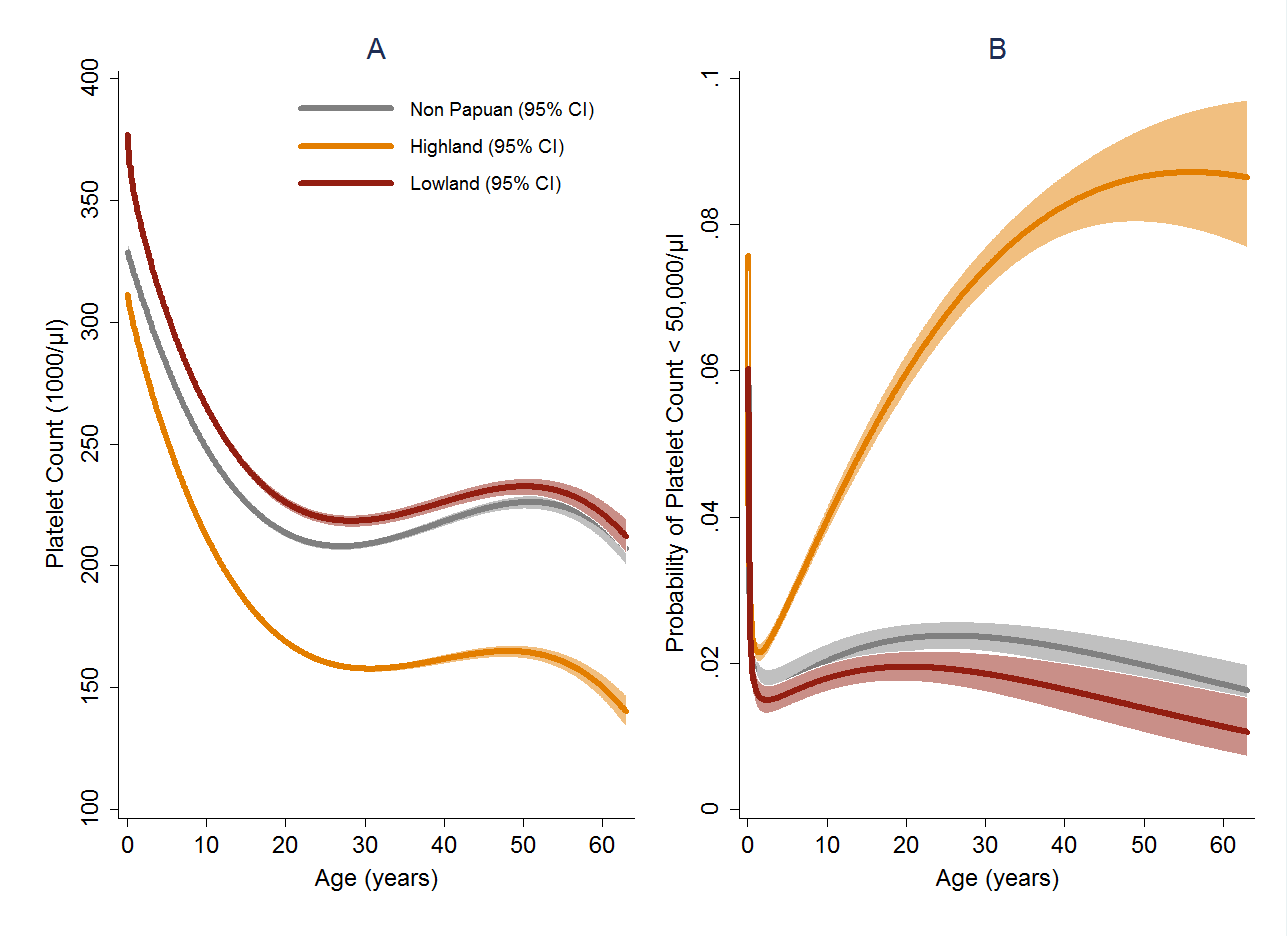

Supplement: Supplementary Data [file supp_jiu487_jiu487supp.docx]
